# Using deep learning to monitor children’s nutritional status for child stunting identification

**DOI:** 10.3389/fped.2026.1725474

**Published:** 2026-08-11

**Authors:** Shijia Luo, Yuan Wang, Rongrong Wu, Zhaoxin Yang, Hui Gao, Jing Yuan, Zhitao Wang

**Affiliations:** 1 School of Computer Science and Technology, Ocean University of China, Qingdao, Shandong, China; 2 Children’s Health Management Center of Handan First Hospital, Handan, China; 3 Handan First Hospital, Handan, China; 4 Handan Maternal and Child Health Hospital, Handan, China

**Keywords:** child stunting identification, children’s nutritional status, deep learning, Intelligent Nutritional Monitoring Model (INMM), tabular health data

## Abstract

**Introduction:**

Children's nutritional health remains a major public health concern, and accurate early identification of growth related risks is essential for timely screening and intervention. This study proposes a deep learning based framework for monitoring children's nutritional status for child stunting identification.

**Methods:**

The task is formulated as a supervised binary classification problem, in which each child is represented by routinely available demographic and anthropometric variables, including age, sex, height, weight, and body mass index. Based on these structured inputs, the proposed Intelligent Nutritional Monitoring Model (INMM) learns discriminative latent representations and predicts whether a child is stunted. The model adopts a compact feature extraction architecture with feature fusion and hierarchical attention, enabling effective modeling of informative interactions among child level attributes while maintaining computational efficiency. To evaluate the proposed method, experiments are conducted on two public health survey datasets, NHANES and NFHS 5, under a unified preprocessing, training, and evaluation protocol.

**Results and Discussion:**

Comparative results against traditional machine learning, mainstream deep learning, lightweight tabular modeling, and transformer based tabular baselines show that the proposed method achieves the strongest overall classification performance on both datasets while preserving a favorable effectiveness efficiency trade off. Additional ablation results further confirm the contribution of feature extraction, fusion, attention, and prediction design to the final performance. These findings indicate that the proposed framework provides an effective and scalable solution for data driven child stunting identification from structured health survey data.

## 1 Introduction

Children’s nutritional status is a critical determinant of physical growth, cognitive development, and long term health. Among nutrition related disorders, child stunting remains one of the most important public health concerns, especially in low and middle income countries where early screening resources are often limited. Conventional assessment of child growth risk mainly relies on manual anthropometric evaluation and survey based review, which are labor intensive, difficult to scale, and often unable to support timely risk identification (1). In this context, data driven approaches provide an important opportunity to improve the efficiency and consistency of nutritional status monitoring. By learning predictive patterns from structured health data, artificial intelligence can support earlier and more standardized identification of children at risk of growth retardation (2).

Early studies in nutritional monitoring were largely based on rule driven systems and expert defined assessment frameworks that mapped reported dietary or health information to nutritional judgments (3). Although these approaches provided initial decision support, they required substantial manual maintenance and were difficult to generalize across changing populations and data environments (4). Subsequent machine learning methods improved flexibility by learning from structured demographic and clinical variables, allowing researchers to model more complex relationships between child characteristics and health outcomes (5). However, many of these methods still depended heavily on manual feature design and often showed limited ability to capture nonlinear interactions among nutritional and anthropometric variables (6). As a result, there remains a need for models that can learn discriminative representations directly from structured child level data while preserving efficiency and applicability in real world screening scenarios (7).

Recent advances in deep learning have further expanded the potential of intelligent nutritional assessment. Neural models have shown strong capacity for representation learning in healthcare prediction tasks and have demonstrated advantages in modeling complex feature interactions beyond conventional linear or tree based methods (8). Nevertheless, practical deployment in child health monitoring requires more than predictive accuracy alone. A useful model should also remain computationally efficient, robust on structured survey data, and suitable for standardized comparison with traditional machine learning and modern tabular learning baselines (9). These considerations are particularly important when the target application is child stunting identification from routine demographic and anthropometric measurements rather than large scale structured sensing environments (10).

It should be noted that this study is not intended as a benchmark oriented state of the art competition, but rather as a task specific investigation of structured data modeling for child stunting identification. Motivated by these observations, this study focuses on using deep learning to monitor children’s nutritional status for child stunting identification. We formulate the task as a supervised binary classification problem based on routinely available child level variables, including age, sex, height, weight, and body mass index. To address this task, we develop an Intelligent Nutritional Monitoring Model (INMM) that learns compact latent representations from structured inputs and performs prediction through feature extraction, feature fusion, and hierarchical attention. This design aims to improve classification performance while maintaining an efficient model structure suitable for practical screening settings. The reason for adopting a deep learning model in this study is that child stunting identification depends not only on individual demographic or anthropometric variables, but also on their potentially nonlinear interactions. While traditional machine learning methods provide strong and important baselines for structured data, they may be limited in learning compact task specific representations in an end to end manner. In contrast, a lightweight deep learning architecture can jointly perform representation learning, feature fusion, and adaptive reweighting within a unified framework. For this reason, we formulate the task under a deep learning setting while also comparing against representative classical and modern tabular baselines to verify whether the additional modeling capacity is justified. Extensive experiments on two public health survey datasets demonstrate the effectiveness of the proposed framework under a unified and reproducible evaluation protocol.

The major contributions of this research can be summarized as follows:

- We reformulate children’s nutritional status monitoring as a supervised deep learning problem for child stunting identification using structured demographic and anthropometric variables.
- We propose the Intelligent Nutritional Monitoring Model (INMM), which combines feature extraction, feature fusion, and hierarchical attention to improve discriminative learning on child level tabular health data.
- We conduct extensive experiments on NHANES and NFHS 5 and show that the proposed method achieves strong classification performance and a favorable effectiveness efficiency trade off compared with representative traditional, deep learning, lightweight, and transformer based tabular baselines.

## 2 Related work

### 2.1 Deep learning in nutritional assessment

Deep learning has become an important approach in nutritional assessment because of its ability to learn discriminative representations from complex health related data (11). Compared with conventional assessment pipelines that rely heavily on manual review of anthropometric indicators and dietary records, deep models provide a more flexible way to capture nonlinear relationships among child characteristics and nutrition related outcomes (12). Existing studies have applied neural networks to growth prediction, nutritional status classification, and health risk screening by using demographic, anthropometric, and clinical variables. In particular, sequence models have been explored for longitudinal growth modeling, while representation learning methods have shown advantages in identifying subtle patterns that are difficult to capture with handcrafted features alone. These developments indicate that deep learning can improve the consistency and predictive power of nutritional assessment systems. However, many existing studies focus on broad nutritional evaluation settings or heterogeneous data modalities, which makes it difficult to compare methods under a clear and practically deployable task definition. In contrast, this work focuses on a more specific and operational problem, namely child stunting identification from structured child level health variables, and evaluates a dedicated deep learning framework under a unified binary classification setting (13).

### 2.2 Learning from structured health and anthropometric data

A large body of work has investigated prediction from structured health data, where each sample is represented by demographic, clinical, or anthropometric attributes rather than images, audio, or free text. For child health assessment, such tabular data are especially important because variables such as age, sex, height, weight, and body mass index are routinely collected in public health surveys and screening programs. Traditional machine learning methods, including linear models, decision tree ensembles, and gradient boosting, have shown strong performance on these inputs because they can model feature interactions with relatively low computational cost (14). More recently, deep tabular models have been introduced to improve representation learning on structured data while preserving end to end optimization and better scalability to complex decision boundaries (15). These models offer a promising direction for child stunting identification, where the target depends on subtle interactions among anthropometric and demographic variables rather than a single threshold rule. Nevertheless, the challenge remains to design a model that is both expressive and efficient, and that can be compared fairly against strong classical and modern tabular baselines. This motivates the present study to adopt a compact deep architecture tailored to structured survey data (16).

### 2.3 Stunting risk identification and predictive child health screening

Child stunting identification is a clinically meaningful prediction task because it supports early screening of growth retardation risk and enables more timely public health response. Existing predictive studies in child health have used survey data, growth measurements, and related socioeconomic indicators to identify children at elevated risk of malnutrition or poor developmental outcomes (17). These studies highlight the importance of standardized data preprocessing, reliable supervision rules, and reproducible evaluation when building screening models for population level use. At the same time, the literature suggests that prediction systems for child health should be assessed not only in terms of discrimination accuracy but also in terms of computational efficiency and robustness, since practical deployment often occurs in settings with limited resources (18). Despite these advances, relatively few studies provide a unified comparison across traditional machine learning methods, mainstream deep learning models, lightweight tabular architectures, and transformer based tabular baselines for the specific task of child stunting identification. Our work addresses this gap by formulating stunting identification as a supervised binary classification problem on structured public health survey data and by evaluating the proposed Intelligent Nutritional Monitoring Model against a comprehensive baseline set under a consistent experimental protocol (19).

## 3 Method

### 3.1 Overview

This section presents the methodological overview of the proposed framework for monitoring children’s nutritional status for child stunting identification. In contrast to a broad sensor-driven or intervention-oriented monitoring framework, the present study focuses on a clearly defined supervised prediction task based on structured child-level health variables. The overall methodology is organized into two tightly connected components: problem formulation and predictive model design. The first component defines the input variables, output label, and learning objective for child stunting identification. The second component introduces the Intelligent Nutritional Monitoring Model (INMM), which is designed to learn effective latent representations from structured demographic and anthropometric data and to produce accurate binary predictions under a computationally efficient architecture. The following subsections describe these components in detail.

The problem formulation specifies each child as one input sample represented by routinely collected variables, including age, sex, height, weight, and body mass index, while the target is a binary stunting label derived under a standardized health criterion. Under this formulation, the task becomes supervised binary classification from structured tabular inputs to stunting risk prediction. To solve this problem, the proposed INMM adopts a compact predictive pipeline consisting of feature extraction, feature integration, attention based weighting, and final classification. The feature extraction stage maps the input variables into latent representations, the fusion stage combines complementary information across learned features, and the hierarchical attention mechanism emphasizes the most informative latent factors for the final decision. The resulting representation is then passed to a prediction head to estimate the probability that a child belongs to the stunted class. This design provides a unified framework that is aligned with the experimental task definition, maintains consistency with the available survey based input variables, and supports accurate as well as efficient child stunting identification in practical screening scenarios.

For clarity and to improve abbreviation consistency in the method description, the main abbreviations and technical terms used in this study are summarized in Table 1.

**Table 1:** Main abbreviations and technical terms used in this study.

Abbreviation/term	Full expression or meaning
INMM	Intelligent Nutritional Monitoring Model
ANIS	Adaptive Nutritional Intervention Strategy
BMI	Body mass index
FC	Fully connected
MLP	Multilayer perceptron
AUROC	Area under the receiver operating characteristic curve
F1 score	Harmonic mean of precision and recall
FLOPs	Floating point operations
NHANES	National Health and Nutrition Examination Survey
NFHS 5	National Family Health Survey 5
WHO	World Health Organization

### 3.2 Preliminaries

The objective of this study is to design a deep learning framework for child stunting identification from structured child level health data. Let $D=\{(x_{i},y_{i}){\}}_{i=1}^{N}$ denote the dataset, where $x_{i}$ represents the input of the ith child and $y_{i}$ is the corresponding binary stunting label. Each input sample is represented as a fixed tabular vector composed of five routinely available variables (Equation 1):

$$x_{i}=[a_{i},g_{i},h_{i},w_{i},b_{i}],$$ (1)

where $a_{i}$ denotes age, $g_{i}$ denotes sex, $h_{i}$ denotes height, $w_{i}$ denotes weight, and $b_{i}$ denotes body mass index. The target label is defined as (Equation 2):

$$y_{i}\in \{0,1\},$$ (2)

where $y_{i}=1$ indicates that the child is stunted and $y_{i}=0$ otherwise. The task is formulated as supervised binary classification, in which a neural prediction function $f_{\theta }$ learns the mapping from structured demographic and anthropometric variables to the stunting label (Equation 3):

$$\min_{\theta }\;L(f_{\theta }(x_{i}),y_{i}),$$ (3)

where L(⋅) denotes the binary classification loss and θ denotes learnable model parameters.

In accordance with the World Health Organization child growth standards, the binary stunting label was defined using the height for age Z score. A child was assigned to the stunted class when the height for age Z score was lower than −2 standard deviations from the WHO reference median. The child was assigned to the non stunted class. The ground truth label used in this study can be written as (Equation 4):

$$y_{i}=\left\{ \begin{array}{cc} 1, & {\mathrm{HAZ}}_{i}<-2,\\ 0, & {\mathrm{HAZ}}_{i}\geq -2, \end{array}\right.$$ (4)

where ${\mathrm{HAZ}}_{i}$ denotes the height for age Z score of the ith child, $y_{i}=1$ denotes stunting, and $y_{i}=0$ denotes non stunting. The supervision signal in both NHANES and NFHS 5 was generated according to this standardized WHO criterion rather than manual annotation, which ensures that the binary classification target is clinically interpretable and reproducible across datasets.

These preliminaries define the input space, output space, supervision rule, and optimization objective used throughout the subsequent model design and experiments. Detailed architectural operations, including branch wise feature extraction, feature fusion, hierarchical attention, and final prediction, are introduced in the following INMM subsection to avoid repeated formulation.

### 3.3 Intelligent Nutritional Monitoring Model (INMM)

**Figure 1:**
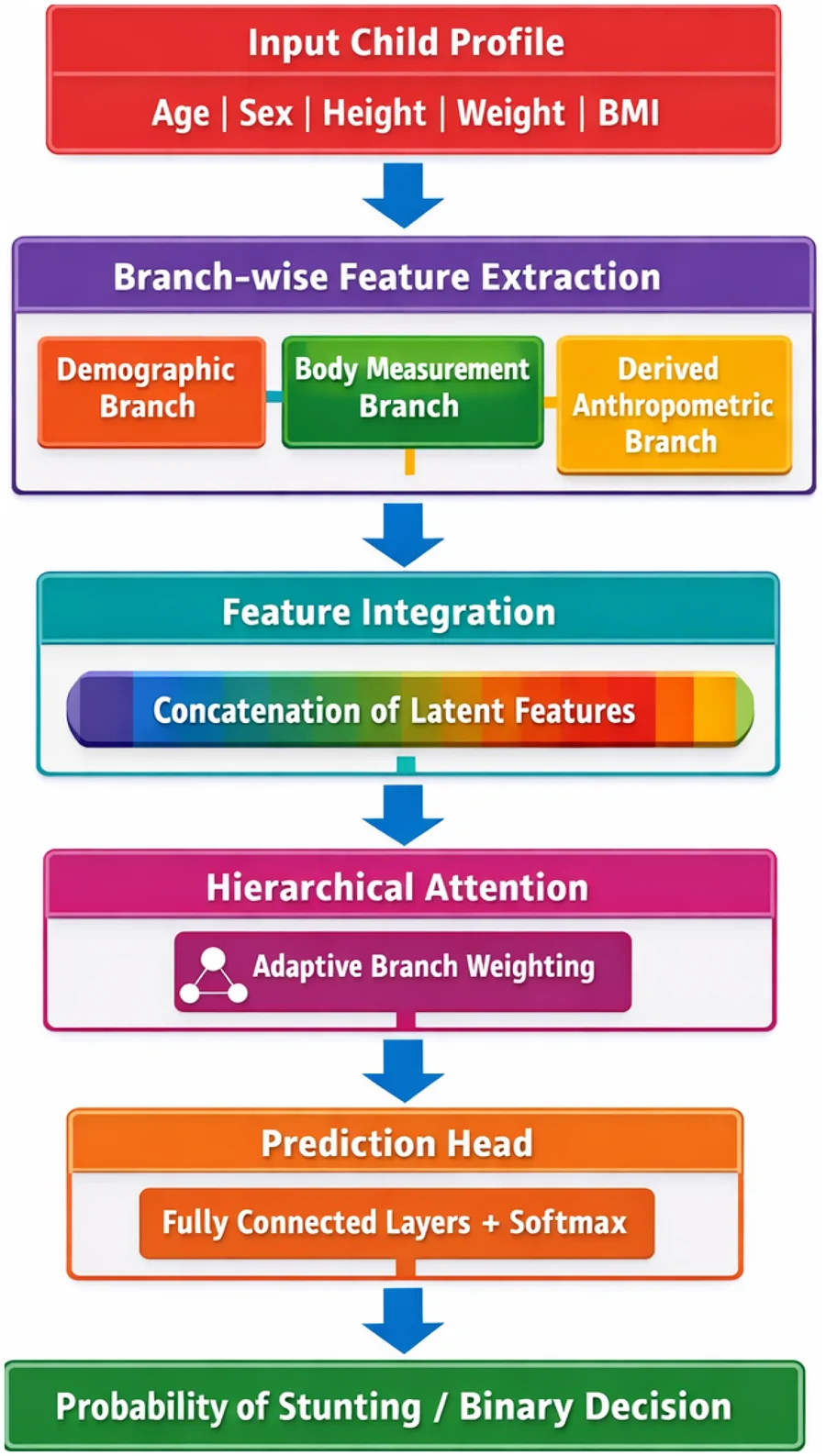
Overview of the Intelligent Nutritional Monitoring Model (INMM) for child stunting identification. The model takes structured child level demographic and anthropometric variables as input, transforms them through branch wise feature extraction, integrates the resulting latent features, applies hierarchical attention to emphasize informative factors, and outputs the probability of the stunted class.

In this section, we present the Intelligent Nutritional Monitoring Model (INMM), a deep learning framework designed for monitoring children’s nutritional status for child stunting identification. In contrast to a broad nutritional assessment or intervention framework, the present model is aligned with the task definition and experimental setting adopted in Section 4, where each child is represented by structured demographic and anthropometric variables. The input consists of age, sex, height, weight, and body mass index, and the output is the probability of the child belonging to the stunted class. As shown in Figure 1, INMM follows a compact predictive pipeline composed of branch wise feature extraction, feature integration, hierarchical attention, and final classification. This design preserves the core idea of intelligent nutritional monitoring while making the model fully consistent with the final supervised binary classification task.

#### 3.3.1 Structured branch-wise feature extraction

**Figure 2:**
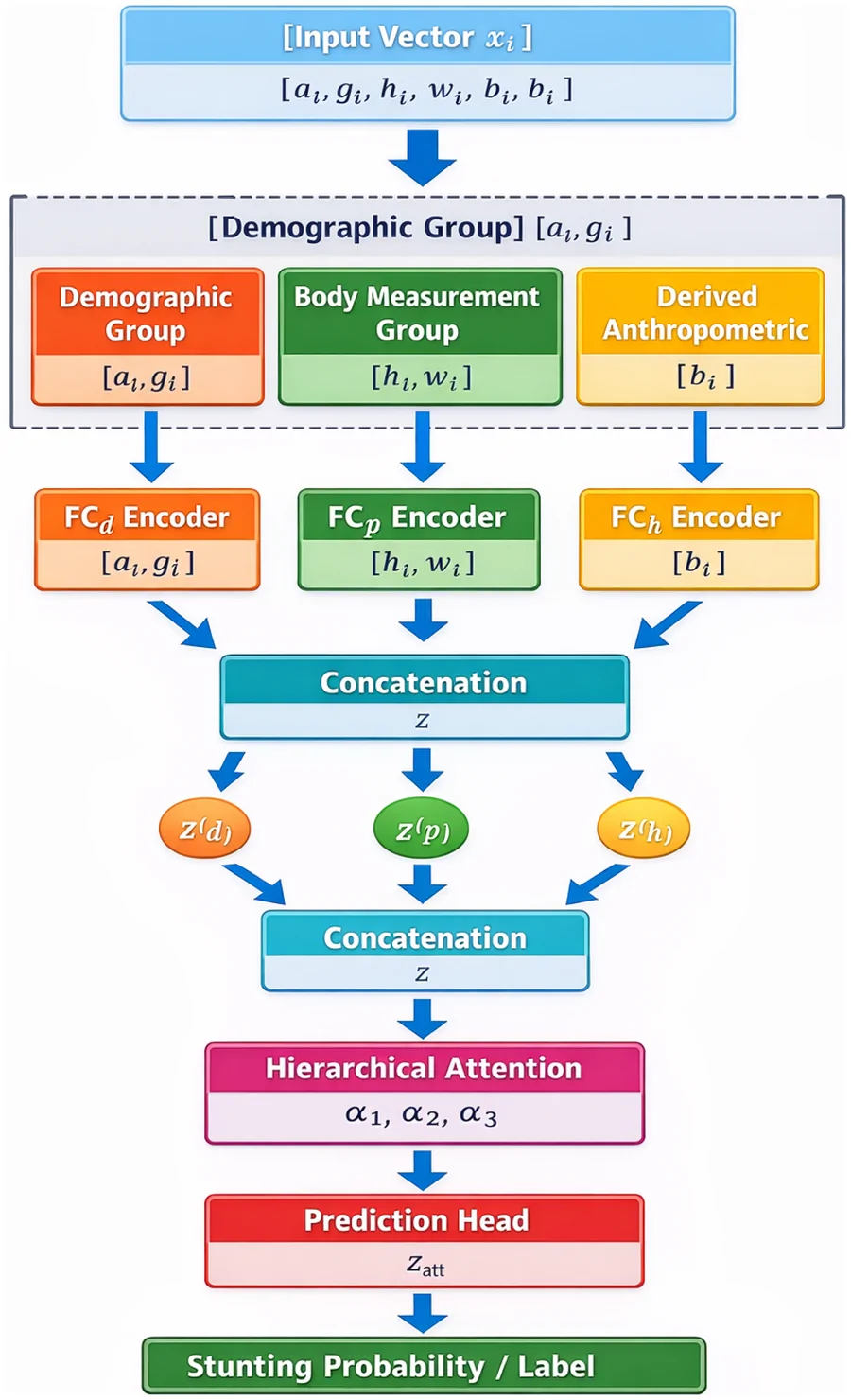
Structured feature extraction and prediction pipeline of INMM. The input child profile is divided into demographic attributes, body measurements, and derived anthropometric information, each of which is encoded by a dedicated fully connected branch. The resulting latent features are fused and reweighted by hierarchical attention before binary stunting prediction.

The INMM begins with a structured feature extraction module that converts the raw tabular input into discriminative latent representations. Although the final data source is structured tabular data rather than heterogeneous sensing modalities, the input variables can still be organized into semantically distinct groups to facilitate representation learning. As shown in Figure 2, the input vector of the ith child is written as (Equation 5):

$$x_{i}=[a_{i},g_{i},h_{i},w_{i},b_{i}],$$ (5)

where $a_{i}$, $g_{i}$, $h_{i}$, $w_{i}$, and $b_{i}$ denote age, sex, height, weight, and body mass index, respectively. We divide this input into three branches (Equation 6):

$$x_{i}^{\left(d\right)}=[a_{i},g_{i}],\;x_{i}^{\left(p\right)}=[h_{i},w_{i}],\;x_{i}^{\left(h\right)}=[b_{i}].$$ (6)

Here, $x_{i}^{\left(d\right)}$ captures demographic context, $x_{i}^{\left(p\right)}$ captures direct body size measurements, and $x_{i}^{\left(h\right)}$ captures derived anthropometric status. Each branch is processed by a branch specific fully connected encoder (Equation 7):

$$z^{\left(d\right)}={\mathrm{FC}}_{d}(x_{i}^{\left(d\right)}),\;z^{\left(p\right)}={\mathrm{FC}}_{p}(x_{i}^{\left(p\right)}),\;z^{\left(h\right)}={\mathrm{FC}}_{h}(x_{i}^{\left(h\right)}),$$ (7)

where ${\mathrm{FC}}_{d}(\cdot )$, ${\mathrm{FC}}_{p}(\cdot )$, and ${\mathrm{FC}}_{h}(\cdot )$ denote nonlinear transformation functions. In this way, INMM learns complementary latent features from different aspects of the child profile before combining them into a unified predictive representation.

The extracted branch wise features are concatenated to form a unified latent representation (Equation 8):

$$z=[z^{\left(d\right)},z^{\left(p\right)},z^{\left(h\right)}].$$ (8)

This fusion step enables the model to jointly encode demographic and anthropometric information in a shared latent space and prepares the representation for the subsequent attention based refinement.

#### 3.3.2 Hierarchical attention mechanism

To emphasize the most informative latent factors for child stunting identification, INMM incorporates a hierarchical attention mechanism. Given the fused representation z, the attention weight for the ith branch is computed as (Equation 9)

$$\alpha_{i}=\frac{\exp\;(w_{i}^{⊤}z)}{\sum_{\;j=1}^{M}\exp\;(w_{j}^{⊤}z)},$$ (9)

where $w_{i}$ is a learnable parameter vector and M=3 is the number of branches. The attended representation is then defined as (Equation 10):

$$z_{\text{att}}=\sum_{i=1}^{M}\alpha_{i}z^{\left(i\right)}.$$ (10)

This mechanism allows the model to adaptively reweight branch level latent features according to their contribution to the final prediction. As a result, INMM can focus more strongly on informative anthropometric or demographic cues when estimating stunting risk, which improves discriminative ability and helps maintain a better balance between positive case detection and false positive control.

#### 3.3.3 Nutritional status prediction module

The final component of INMM is the nutritional status prediction module, which maps the attended representation to the binary output space. This is implemented by a fully connected prediction head followed by a softmax activation (Equation 11):

$$\hat{y}=\mathrm{Softmax}(Wz_{\text{att}}+b),$$ (11)

where W and b are learnable parameters, and $\hat{y}$ denotes the predicted probability distribution over the two classes. Under this formulation, the model outputs the probability that a child is stunted given the structured demographic and anthropometric input. The overall INMM therefore preserves the original design logic of feature extraction, feature fusion, attention weighting, and prediction, while remaining fully consistent with the final experimental task of child stunting identification. This design is compact, reproducible, and suitable for efficient nutritional status monitoring in practical child health screening scenarios.

### 3.4 Adaptive nutritional intervention strategy

**Figure 3:**
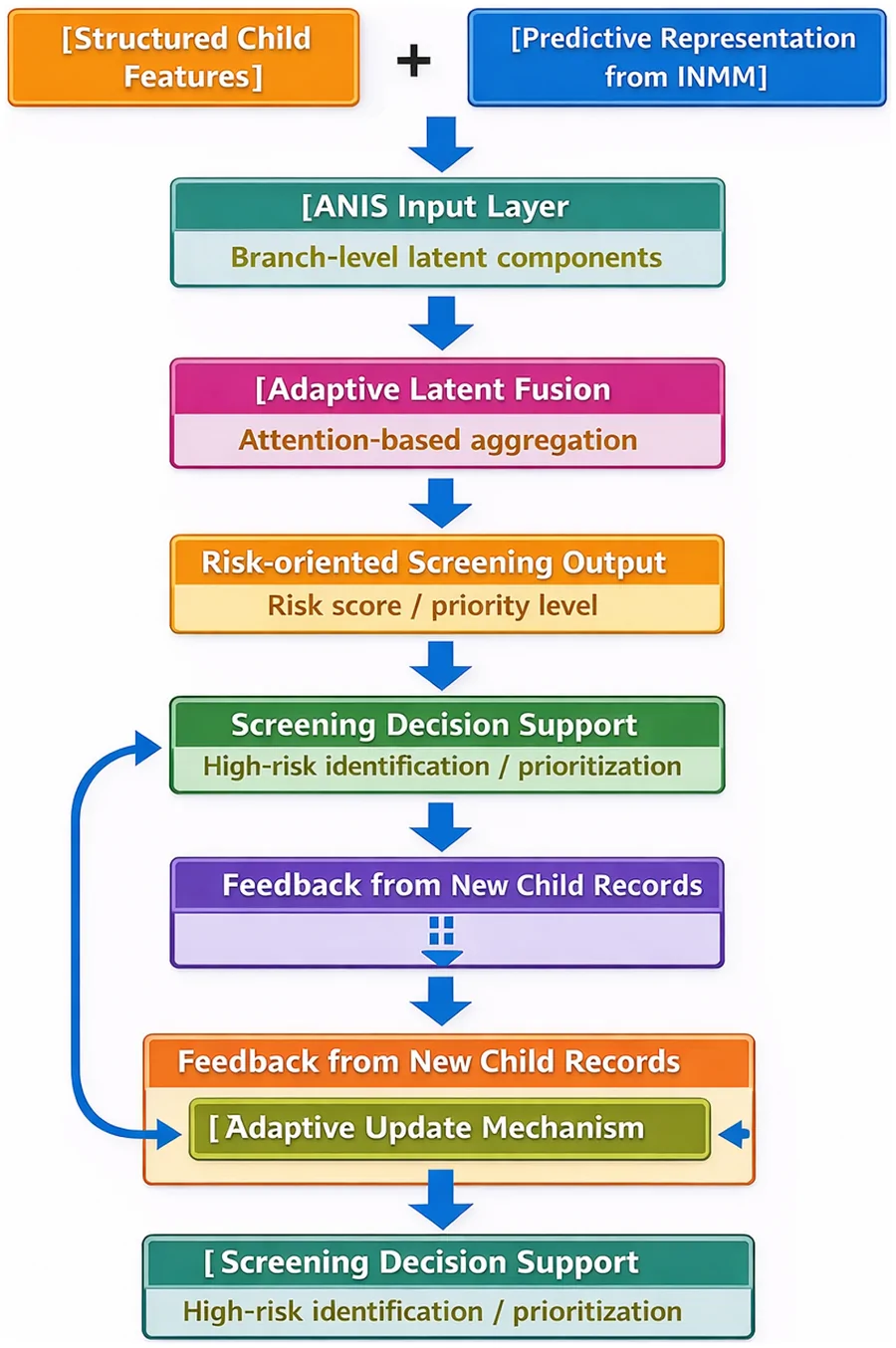
Overview of the Adaptive Nutritional Intervention Strategy (ANIS) as a post prediction decision support extension for child stunting identification. The strategy takes structured child level features and predictive representations from INMM, aggregates informative latent factors, estimates screening oriented risk scores, and supports feedback aware refinement when new records are observed.

This subsection presents the Adaptive Nutritional Intervention Strategy (ANIS), which serves as a decision support extension built on top of the predictive outputs of INMM. In this work, ANIS is not formulated as a reinforcement learning system or a treatment policy optimization framework, and it is not evaluated as an independent intervention module. Instead, it is designed as a lightweight decision support extension that operates on the predictive outputs of INMM to provide risk oriented interpretation and prioritization. Instead, it is designed as an adaptive risk interpretation and prioritization strategy for child stunting identification. Given the structured child level input and the prediction score produced by INMM, ANIS refines the representation of stunting risk, supports priority ranking of children who may require closer screening attention, and provides a possible mechanism for model side adaptation when new child records become available. As shown in Figure 3, ANIS operates after the predictive stage and uses latent feature aggregation, screening oriented risk scoring, and feedback aware refinement to improve the practical usability of the overall framework.

#### 3.4.1 Structured data fusion mechanism

**Figure 4:**
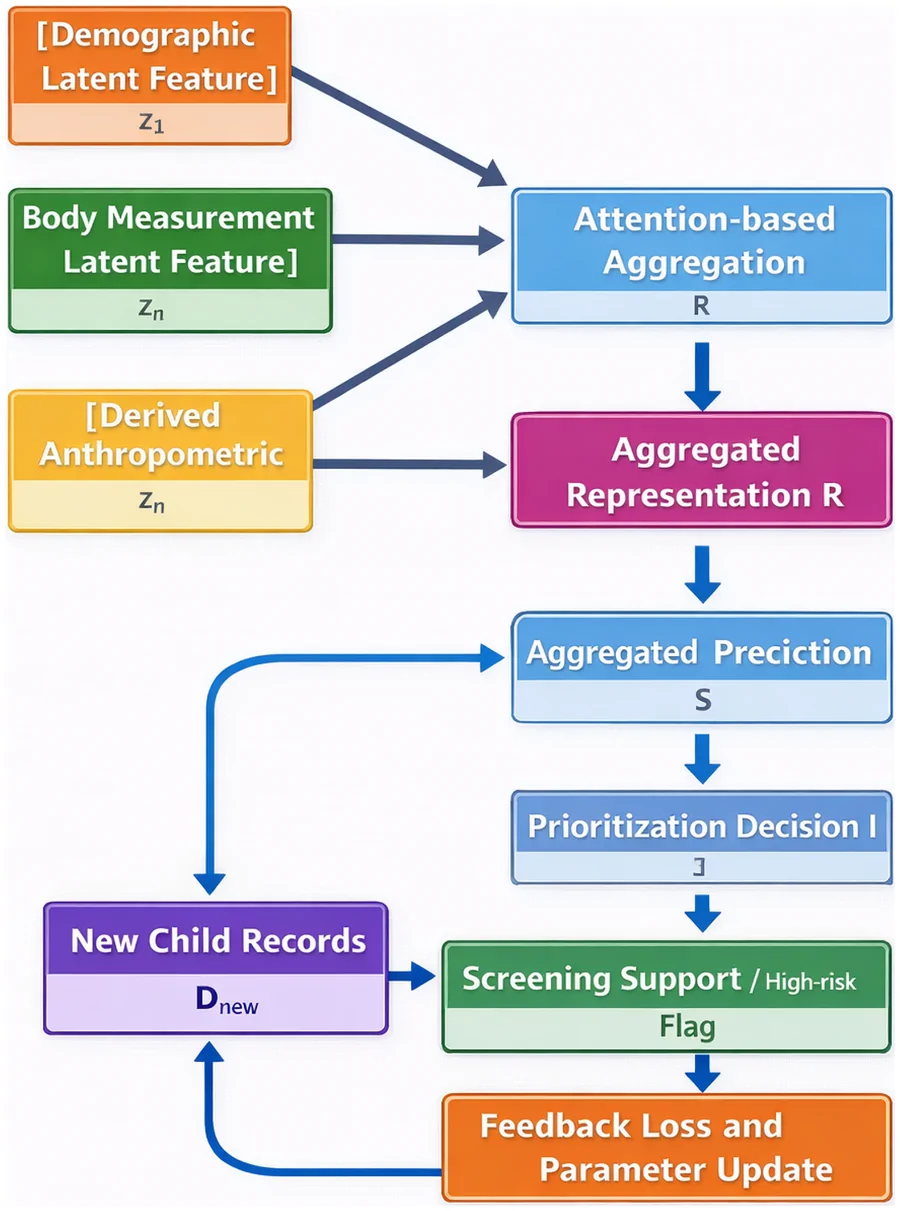
Structured decision support pipeline of ANIS. Branch level latent features from demographic attributes, body measurements, and derived anthropometric information are adaptively fused through attention based aggregation, converted into screening oriented risk scores, and optionally refined through a feedback aware update mechanism.

The core of ANIS is an adaptive latent fusion mechanism that operates on the structured representations extracted from the child profile. Although the final task does not use heterogeneous sensing modalities, the model still produces multiple branch level latent features corresponding to demographic attributes, body measurements, and derived anthropometric status. These complementary latent components are treated as distinct information sources for downstream screening oriented decision support. As shown in Figure 4, let (Equation 12):

$$X=\{x_{1},x_{2},\ldots ,x_{n}\}$$ (12)

denote the set of branch level inputs or latent components derived from the structured child profile. Here, n denotes the number of branch level components involved in the structured latent aggregation process.

The system first transforms these components through feature extraction functions to obtain latent representations (Equation 13):

$$z_{i}=f_{\text{extract}}(x_{i};\theta_{i}),$$ (13)

where $f_{\text{extract}}$ is the branch specific transformation parameterized by $\theta_{i}$. To capture the interdependence among these latent components, ANIS employs an attention based aggregation mechanism. Let A denote the attention matrix, in which each element $a_{ij}$ quantifies the relevance of component j to component i. The attention scores are computed as (Equation 14):

$$a_{ij}=\frac{\exp\;(\phi (z_{i},z_{j}))}{\sum_{k=1}^{n}\exp\;(\phi (z_{i},z_{k}))},$$ (14)

where ϕ(⋅,⋅) is a learnable compatibility function. The aggregated representation is then obtained by (Equation 15):

$$r_{i}=\sum_{\;j=1}^{n}a_{ij}z_{j}.$$ (15)

This mechanism allows ANIS to integrate complementary evidence across branch level latent features and to produce a more robust representation for downstream screening oriented risk interpretation.

The set of aggregated branch representations is denoted by $R=\{r_{1},r_{2},\ldots ,r_{n}\}$, which is further used as the input to the downstream screening oriented prediction module.

#### 3.4.2 Dynamic nutritional status prediction

Based on the aggregated representation, ANIS produces a risk oriented prediction that complements the binary classifier in INMM. The system estimates a stunting related screening output (Equation 16):

$$S=f_{\text{\textbackslash{},predict}}(R;\Theta ),$$ (16)

where R denotes the aggregated latent representation and Θ represents the parameters of the prediction module. In the context of this study, S is interpreted as an auxiliary risk output associated with the probability and relative priority of child stunting rather than as a multidimensional intervention target. To support adaptive screening prioritization, we define a target aware optimization objective (Equation 17):

$$L=\|S-T{\|}^{2}+\lambda \cdot C(I),$$ (17)

where T denotes the desired screening target, I denotes the prioritization decision, C(I) encodes practical constraints on the decision process, and λ is a regularization coefficient. The decision variable is updated iteratively by (Equation 18):

$$I^{\left(t+1\right)}=I^{\left(t\right)}-\eta \nabla_{I}L,$$ (18)

where η is the learning rate. Under this formulation, ANIS can be understood as a lightweight adaptive strategy that converts predictive outputs into screening oriented decision support while remaining consistent with the final binary stunting identification task. This formulation is used to conceptually describe an iterative refinement of prioritization decisions, rather than an actual optimization procedure implemented in the experiments.

#### 3.4.3 Adaptive feedback loop for model update

To improve adaptability under evolving survey data, ANIS incorporates a feedback aware update mechanism. Let $D_{\text{new}}$ denote newly available child records and let M denote the current model parameters. The update rule is defined as (Equation 19):

$$M^{\left(t+1\right)}=M^{\left(t\right)}+\gamma \nabla_{M}L_{\text{\textbackslash{},feedback}}(D_{\text{new}},M),$$ (19)

where γ is the feedback learning rate and $L_{\text{\textbackslash{},feedback}}$ is the adaptation loss. This mechanism enables the model to refine its risk estimation behavior when new structured child records become available, thereby improving robustness to distributional variation across screening populations.

For clarity, the main notations in the above extension are summarized as follows. S denotes the screening oriented risk output, T denotes the desired screening target, I denotes the prioritization decision variable, C(I) denotes the constraint term associated with the decision process, λ denotes the regularization coefficient, and η denotes the update step size for decision refinement. In the feedback aware update equation, $D_{\mathrm{new}}$ denotes newly available child records, $M^{\left(t\right)}$ denotes the model parameters at update step t, γ denotes the feedback learning rate, and $L_{\mathrm{feedback}}$ denotes the adaptation loss used for model refinement.

In the present study, this feedback formulation is included as an adaptive extension of the overall framework rather than as the primary object of empirical evaluation, while the core experiments remain focused on the supervised binary classification performance of child stunting identification. In this way, ANIS preserves the original methodological idea of adaptive nutritional decision support but is expressed in a form that is coherent with the final experimental scope and the structured survey based prediction setting. It should be emphasized that the ANIS component is not empirically evaluated as an intervention or decision policy in this study. The current experiments focus exclusively on supervised binary classification performance. ANIS is included as a conceptual extension to illustrate how predictive outputs could be integrated into a screening oriented decision support process in future work.

## 4 Experiments

### 4.1 Task definition

In this study, the term nutritional status is operationalized as child stunting status under the WHO child growth standards, so that the final prediction target is a binary indicator of whether a child is classified as stunted or non stunted. The experimental task is defined as child stunting identification under a supervised binary classification setting. Each sample corresponds to one child and is represented by a fixed tabular input vector X=[a,g,h,w,b], where a denotes age, g denotes sex, h denotes height, w denotes weight, and b denotes body mass index. The target output is a binary label Y∈{0,1} indicating whether the child is stunted. In this study, the model learns a mapping $f_{\theta }\;:\;X\to Y$ from basic anthropometric and demographic variables to the stunting decision. This formulation is consistent with the experimental objective of identifying developmental retardation risk from routinely available child level measurements, and it converts the original health assessment problem into a well defined prediction task that is directly measurable with classification metrics.

The supervision signal is derived from standardized health survey data rather than manual annotation during model development. Child records are collected from NHANES and NFHS 5, and the ground truth stunting labels are generated according to the WHO child growth standards using the corresponding child anthropometric measurements. The label source is rule based expert supervision grounded in established clinical and public health criteria. Under this setting, the training process optimizes model parameters on labeled child samples, while the evaluation stage measures how accurately the predicted label matches the WHO derived stunting status. This design ensures that the task definition, input variables, and supervision source remain fully aligned with the experimental protocol and support fair comparison across all baseline and proposed models.

### 4.2 Dataset and data preprocessing

#### 4.2.1 Datasets

We conduct experiments on two public health survey datasets, NHANES (20) and NFHS 5 (21), both of which provide structured child level records for nutritional and anthropometric assessment. NHANES is a publicly released national health and nutrition survey from the United States, and NFHS 5 is a publicly released national family health survey from India. The selection of NHANES and NFHS 5 is consistent with the objective and scope of this study. The objective of the paper is to develop and evaluate a supervised learning framework for child stunting identification from routinely available structured demographic and anthropometric variables. Both datasets provide child level health survey records containing variables that can be aligned with this objective, including age, sex, height, weight, and body mass index. These variables are also directly related to the WHO based definition of stunting through height for age assessment. Therefore, the two datasets are relevant to the target task and suitable for evaluating whether the proposed model can identify stunting status from compact and routinely collected child growth information. The use of two public survey datasets also supports the intended scope of the study. NHANES and NFHS 5 represent different population sources, survey systems, and nutritional contexts, which makes them useful for examining the consistency of the proposed method under separate public health data environments. At the same time, the experiments do not combine heterogeneous records into a single mixed population model. The same preprocessing pipeline, feature definition, label generation rule, and evaluation protocol are applied to each dataset separately. This design ensures that the dataset use remains aligned with the paper objective, while also allowing a fair assessment of model performance across two independent sources of child anthropometric data. Records without the required child level variables or without valid WHO based stunting labels are excluded, so the final retained samples are restricted to cases that directly support the supervised binary stunting identification task. For both datasets, the task is formulated as binary child stunting identification, so the label space contains two classes: stunted and non stunted. Each retained sample consists of five input variables, namely age, sex, height, weight, and BMI, together with one binary target label. The supervision signal is generated by applying the WHO child growth standards to the anthropometric measurements of each child, which yields a rule based label under a unified clinical criterion rather than manual annotation. During preprocessing, we retain only child records with complete values for all five input variables and the derived stunting label, remove entries with missing demographic or anthropometric fields, and discard records that do not satisfy the child sample requirement of the study. To ensure consistency across the two datasets, all variables are converted into a unified tabular format, categorical sex values are numerically encoded, and continuous features are standardized using statistics computed from the training split only. Because the prediction target is assigned at the individual child level, the data are split at the sample level with no overlap between training, validation, and test sets. We adopt a stratified random split with a ratio of 8:1:1 for each dataset so that the class distribution remains stable across the three subsets. Detailed dataset statistics after preprocessing and stratified splitting are summarized in Table 2. After preprocessing and sample filtering, the final number of retained child records was 3,960 for NHANES and 9,850 for NFHS 5. In NHANES, the retained dataset contained 1,662 stunted samples and 2,298 non stunted samples, while in NFHS 5, the retained dataset contained 4,128 stunted samples and 5,722 non stunted samples. Using the stratified 8:1:1 split, the NHANES dataset was divided into 3,168/396/396 samples for training, validation, and testing, respectively, and the NFHS 5 dataset was divided into 7,880/985/985 samples. These statistics provide a clear description of the effective dataset size used in the final experiments. This protocol yields a consistent experimental setting for cross model comparison on two independent population sources while preserving the same feature definition, label generation rule, filtering standard, and evaluation pipeline.

**Table 2:** Dataset statistics after preprocessing and stratified splitting.

Dataset	Total	Stunted	Non stunted	Train	Validation	Test
NHANES	3,960	1,662	2,298	3,168	396	396
NFHS 5	9,850	4,128	5,722	7,880	985	985

To further clarify the population scope and age range handling, detailed dataset information is summarized in Table 3. Since NHANES and NFHS 5 are collected from different national populations and use different survey systems, age information was first converted into a unified month based representation whenever necessary. To ensure that the supervised learning task was comparable across datasets, only child records within the common eligible age range of 0 to 59 months were retained. Records outside this shared age range were excluded before model training and evaluation. This restriction avoids introducing age distribution mismatch caused by different survey inclusion criteria and ensures that the WHO based height for age Z score label is applied within a consistent child growth assessment range across both populations. After this age range alignment, the same five structured input variables, label generation rule, preprocessing pipeline, and stratified split protocol were applied to NHANES and NFHS 5. Although the same age range was used for both datasets, applying one modeling framework to NHANES and NFHS 5 remains challenging because the two populations differ substantially in demographic background, nutritional environment, socioeconomic conditions, healthcare access, and survey design. These differences may affect the distributions of age, height, weight, BMI, and stunting prevalence, and may also change the relationship between anthropometric variables and the final stunting label. The age range alignment in this study should be interpreted as a necessary preprocessing step for fair cross dataset comparison, rather than as evidence that the two populations are fully homogeneous. The model was trained and evaluated separately under the same protocol on each dataset, and further external validation, population specific calibration, and subgroup analysis are still needed before applying a single fixed model across demographically distinct child populations.

**Table 3:** Detailed dataset information and age range handling after preprocessing.

Dataset	Population source	Retained samples	Age representation	Retained age range	Input variables
NHANES	United States national health and nutrition survey	3,960	Converted to months	0 to 59 months	Age, sex, height, weight, BMI
NFHS 5	India national family health survey	9,850	Converted to months	0 to 59 months	Age, sex, height, weight, BMI

#### 4.2.2 Data preprocessing

The preprocessing pipeline is designed for structured child level tabular data from NHANES and NFHS 5 and follows a strict sequence of cleaning, feature alignment, and normalization. Duplicate records are removed to ensure that each child contributes only one sample to the analysis. Missing value cleaning is performed by discarding any record with an empty field in age, sex, height, weight, BMI, or the derived stunting label, so that all retained samples contain a complete input output pair. Third, abnormal records are filtered using explicit physiological consistency rules: samples with non positive age, height, weight, or BMI are removed, and BMI is recomputed from height and weight to verify internal consistency; records with inconsistent BMI values are excluded. After cleaning, all retained variables are transformed into a unified tabular representation across the two datasets. Sex is converted into a fixed numerical identity code, and the remaining continuous variables, including age, height, weight, and BMI, are standardized by z score normalization using the mean and standard deviation estimated from the training split only; the same statistics are then applied to the validation and test splits to avoid information leakage. This step aligns feature scales and stabilizes optimization for all compared models, especially MLP, TabNet, and FT Transformer. No tokenization, temporal windowing, stride based sampling, frame selection, or data augmentation is used, because the final task is defined on static child level records rather than sequential, visual, or acoustic inputs. No external feature extractor is introduced, and all experiments are conducted directly on the cleaned and standardized demographic and anthropometric variables. This preprocessing protocol ensures that the final model input is compact, numerically consistent, and fully matched to the supervised binary classification objective of child stunting identification.

In the preprocessing stage, sex was encoded as a binary numerical variable using a consistent mapping across both datasets. The two sex categories were mapped to 0 and 1 before model training, and the same encoding rule was applied to NHANES and NFHS 5 to ensure that the input representation remained comparable across datasets. Although this unified encoding and preprocessing protocol supports fair cross dataset evaluation, applying the same model to NHANES and NFHS 5 still involves challenges caused by demographic and population heterogeneity. NHANES and NFHS 5 represent substantially different population contexts in terms of ethnicity, socioeconomic background, nutritional environment, healthcare access, growth patterns, and survey design. These differences may lead to distribution shifts in the relationships among age, sex, height, weight, BMI, and stunting status. As a result, a model trained or optimized under one population may not necessarily maintain the same calibration, decision threshold, or error profile when transferred to another demographically distinct population. In this study, this issue was partially addressed by applying the same feature definition, label generation rule, preprocessing pipeline, and evaluation protocol to both datasets. Further external validation, population specific calibration, and subgroup performance analysis are still needed before the same model can be reliably deployed across heterogeneous child populations.

The input variables were limited to age, sex, height, weight, and BMI for two main reasons. These variables are consistently available in both NHANES and NFHS 5 and can be directly aligned under the same preprocessing and label generation protocol. This design ensures that the proposed model is evaluated using a compact and comparable structured input space across two demographically distinct datasets. The selected variables are routinely collected in child growth assessment and are closely related to the WHO based anthropometric definition of stunting, making the model practical for low resource screening scenarios where richer household or socioeconomic information may be incomplete, unavailable, or inconsistently recorded. Richer variables such as maternal education, household wealth, dietary information, sanitation conditions, and healthcare access are indeed important determinants of child stunting. These variables were not included in the current model because their definitions, coding schemes, availability, and missingness patterns differ substantially between NHANES and NFHS 5. Directly incorporating such heterogeneous variables could introduce dataset specific biases, reduce cross dataset comparability, and make it difficult to distinguish architectural performance from the effect of unequal feature availability. This study intentionally focuses on a minimal set of harmonized demographic and anthropometric variables to establish a reproducible baseline framework for structured tabular stunting identification. Future work will extend the proposed framework by incorporating socioeconomic, maternal, dietary, household, and environmental variables after careful feature harmonization, missing data handling, and subgroup validation. Such extensions may improve predictive completeness and provide more comprehensive risk profiling, but they require dedicated analysis beyond the compact input setting considered in the present study.

### 4.3 Evaluation metrics and baseline

#### 4.3.1 Metrics definition

We evaluate child stunting identification with four primary effectiveness metrics and two auxiliary efficiency metrics. The primary metrics are AUROC, F1 score, Sensitivity, and Specificity. AUROC measures the ranking quality of the predicted stunting probability over all decision thresholds and reflects the model’s overall discrimination ability between stunted and non stunted children. F1 score is the harmonic mean of precision and recall and is used to summarize the balance between positive prediction accuracy and positive case coverage under binary classification. Sensitivity measures the true positive rate and quantifies how well the model detects children with stunting, which is critical because missed positive cases directly reduce screening utility. Specificity measures the true negative rate and reflects the ability to avoid false alarms on non stunted children. To predictive performance, we report Params and FLOPs as efficiency metrics. Params measures the total number of trainable parameters and characterizes model size and storage cost. FLOPs measures the computational complexity of one forward inference pass and reflects deployment efficiency. Together, these six metrics provide a complete evaluation from both clinical decision quality and resource consumption perspectives for structured child level classification. Because the task in this study is supervised binary child stunting identification, the evaluation metrics should reflect both classification effectiveness and practical screening utility. In particular, AUROC is used to measure overall discrimination across thresholds, F1 score is used to reflect the balance between precision and recall, Sensitivity is used to quantify the ability to detect stunted children, and Specificity is used to evaluate the ability to avoid false alarms on non stunted children. Since practical deployment may occur in resource constrained screening settings, Params and FLOPs are further reported to characterize model size and computational complexity.

#### 4.3.2 Evaluation protocol

The evaluation protocol follows a standard offline supervised classification setting on fixed tabular child records. There is no Top K retrieval setting, no IoU threshold, and no PCKh threshold, because the task is binary classification rather than ranking, detection, or pose estimation. Each model receives the same five input variables, namely age, sex, height, weight, and BMI, and outputs a binary stunting prediction together with a classification score. Performance is evaluated on the held out test split after model selection on the validation split, and the same preprocessing rules, feature definition, and label generation criterion are used for all compared methods. The data split is performed at the individual sample level with stratified random partitioning, so there is no identity overlap between training, validation, and test subsets. Since the retained features are static child level attributes rather than temporal trajectories, no time based split or temporal consistency protocol is required. The entire study is conducted under an offline evaluation regime, where models are trained on labeled survey data and assessed on unseen test samples without user interaction or online feedback. This protocol ensures direct and fair comparison across traditional machine learning methods, deep tabular models, and the proposed method under the same binary stunting identification objective.

#### 4.3.3 Statistical settings

To ensure robust and reproducible comparison, all experiments are repeated for N=5 independent runs with different random seeds. For each metric, we report the mean and standard deviation across the five runs in the form of mean ± standard deviation. This setting is applied consistently to all primary metrics, including AUROC, F1 score, Sensitivity, and Specificity, as well as to the auxiliary efficiency measurements when repeated estimation is required under the same training pipeline. The use of multiple random seeds reduces the influence of random initialization, stochastic mini batch ordering, and training variance, which is particularly important for neural models trained with Adam optimization. To assess whether the proposed method achieves statistically reliable improvement over each baseline, we perform a two sided paired t test on the run level results. The significance threshold is fixed at p<0.05. Pairing is conducted run by run under aligned experimental settings so that the comparison isolates model differences rather than data protocol variation. This statistical configuration provides a stable basis for reporting central tendency, dispersion, and significance, and it supports rigorous comparison across methods with different inductive biases, parameter scales, and computational budgets.

#### 4.3.4 Baseline

We compare the proposed method against six baselines that cover traditional machine learning, mainstream deep learning, lightweight deep tabular modeling, and state of the art table representation learning. Logistic Regression serves as a linear traditional baseline and evaluates whether the task can be solved by a simple discriminative decision boundary on demographic and anthropometric variables. Random Forest is included as a classical ensemble baseline that captures nonlinear feature interactions through bagged decision trees. XGBoost is used as a strong gradient boosting baseline and represents a highly competitive method for structured tabular prediction. MLP serves as a mainstream deep learning baseline and provides a standard fully connected neural architecture for supervised classification on normalized tabular inputs. TabNet is selected as a lightweight deep tabular baseline because it combines sequential feature selection with efficient end to end learning under moderate computational cost. FT Transformer is adopted as the table specific state of the art baseline and represents the strongest transformer style architecture in the comparison set. All baselines are trained and evaluated on the same input variables, preprocessing pipeline, data split, and statistical protocol so that the comparison focuses solely on differences in modeling capacity and inductive structure.

### 4.4 Implementation details

All experiments are conducted on a single workstation running Ubuntu 22.04 LTS with an Intel Xeon Silver 4,314 CPU, one NVIDIA RTX 4,090 GPU, and 128 GB RAM. The software environment is built with Python 3.10, PyTorch 2.1.0, CUDA 12.1, and cuDNN 8.9. NumPy 1.26, scikit learn 1.3, XGBoost 2.0, and pandas 2.1 are used for data processing, baseline construction, and metric computation. The hardware used for experimentation was selected to support efficient training, repeated runs, and fair comparison among all baseline methods under a unified environment, rather than to define the deployment requirement of the proposed framework. The proposed INMM is a compact tabular model with 0.061M trainable parameters and 0.129M FLOPs, so the inference stage has substantially lower computational demand than the training stage. In practical child screening scenarios, the trained model can run on ordinary CPU based hardware, including a standard desktop computer, a low cost laptop, or an entry level edge computing device. This is because each prediction only uses five structured child level variables, namely age, sex, height, weight, and BMI. High performance GPU resources are mainly beneficial for centralized training, repeated validation, and periodic model updating, whereas routine field inference can be conducted with much lower computational resources. A feasible deployment strategy for resource limited regions is therefore to train or update the model at a central facility and distribute the trained parameters and preprocessing statistics to local health sites for CPU based single sample or small batch inference. Under this setting, the minimum practical hardware requirement is a general purpose CPU with sufficient memory to load the lightweight network and the corresponding preprocessing parameters.

The proposed model is trained for 100 epochs with a batch size of 64, which is directly aligned with the training configuration specified in the method summary. Adam is used as the optimizer with $\beta_{1}=0.9$ and $\beta_{2}=0.999$. The initial learning rate is set to $1\times 10^{-3}$, and the weight decay is fixed at $1\times 10^{-4}$. A linear warm up scheduler is applied during the first 10 epochs, followed by cosine annealing over the remaining epochs. The random seed is fixed to 42 for each run and changed across the five repeated runs according to a predefined seed list. Mixed precision training is enabled to improve computational efficiency, and dropout is applied to fully connected layers with a rate of 0.5. Early stopping is not used, because all methods are trained under the same fixed epoch protocol and the final model is selected according to validation performance. The model configuration follows the method summary while adapting the architecture to the final tabular stunting classification task. Since the experimental input is a five dimensional child level feature vector containing age, sex, height, weight, and BMI, the network uses a compact three branch feature encoder that maps the input into a unified latent space before prediction. The hidden dimension of each branch is set to 64, and the output dimension of each branch is also fixed at 64, producing three latent representations that are concatenated into a 192 dimensional fused feature. The hierarchical attention module is implemented with three learnable attention scores followed by softmax normalization to generate branch weights and compute the attended representation. The prediction head consists of two fully connected layers with hidden sizes 128 and 64, ReLU activation, and a final softmax classifier for binary prediction. To preserve the implementation spirit of the original method summary, the feature extraction stage, fusion stage, attention stage, and prediction stage are kept explicit in the final network design. For reproducibility and stability on structured survey data, all linear layers are initialized with Xavier uniform initialization, and layer normalization is inserted before the final prediction head. This configuration remains compact, matches the tabular input structure, and is consistent with the reported training strategy based on PyTorch, Adam, dropout, warm up, and fixed length supervised optimization. For fairness, all baseline methods and the proposed model are trained and evaluated on the same data splits, preprocessing pipeline, input variables, and hardware environment. Hyperparameters of all compared methods are tuned on the validation set only, and the final results are reported on the held out test set under the same repeated run statistical protocol. This setting ensures that performance differences are attributable to model design rather than inconsistency in data access, computation budget, or evaluation procedure.

For reproducibility, the detailed network implementation is specified as follows. Each of the three branch encoders is implemented as a single fully connected layer that maps its corresponding input subvector to a 64 dimensional latent representation, followed by a ReLU activation and dropout with rate 0.5. Concretely, the demographic branch maps the 2 dimensional input $\left[a_{i},g_{i}\right]$ to 64 dimensions, the body measurement branch maps the 2 dimensional input $\left[h_{i},w_{i}\right]$ to 64 dimensions, and the anthropometric status branch maps the 1 dimensional input $\left[b_{i}\right]$ to 64 dimensions. The three resulting branch representations are concatenated into a 192 dimensional fused feature vector. The hierarchical attention module uses three learnable branch specific parameter vectors to compute branch level attention scores from the fused representation, and softmax normalization is then applied across the three branches to obtain branch weights. The attended representation is computed as the weighted sum of the three branch features. The prediction head consists of two fully connected layers with hidden sizes 128 and 64, respectively. Each hidden layer is followed by ReLU activation and dropout, and layer normalization is applied before the final softmax classifier. During training, the model is optimized using the standard cross entropy loss for binary classification. To improve reproducibility across repeated runs, we use five predefined random seeds: 42, 52, 62, 72, and 82. All other training settings are kept identical across runs and across all compared methods unless the baseline implementation requires task specific adaptation. All results are reported as mean ± standard deviation over five independent runs under a unified experimental protocol, which improves the reliability of the comparison. The above specifications provide a complete and reproducible description of the input features, preprocessing procedure, model configuration, training strategy, and evaluation protocol used in this study.

### 4.5 Results and discussion

#### 4.5.1 Comparative experiments

We conduct comparative experiments on the two public health survey datasets defined in the experimental plan, namely NHANES and NFHS 5, under the same supervised binary classification setting for child stunting identification. The comparison covers six baselines, including Logistic Regression, Random Forest, XGBoost, MLP, TabNet, and FT Transformer, which together represent traditional machine learning, mainstream deep learning, lightweight deep tabular modeling, and state of the art tabular architectures. The evaluation follows the predefined protocol and reports four primary metrics, including AUROC, F1 score, Sensitivity, and Specificity, together with two efficiency metrics, namely Params and FLOPs. All models are trained and evaluated on the same input variables, preprocessing pipeline, data split strategy, repeated run protocol, and computing environment. Hyperparameters are selected on the validation set only, and the final results are reported on the held out test set as mean ± standard deviation over five runs. This design ensures reproducibility, fairness, and statistical reliability, and it allows the observed performance differences to be attributed to model design rather than inconsistent experimental conditions.

The results in Tables 4–6 show that our proposed method achieves the best overall performance on both public health survey datasets while maintaining a moderate computational cost. On NHANES, the proposed method reaches an AUROC of 0.961±0.003 and an F1 score of 0.892±0.005, outperforming the strongest baseline, FT Transformer, by 1.1 percentage points in AUROC and 1.3 percentage points in F1 score. It also improves Sensitivity from 0.867 to 0.883 and Specificity from 0.918 to 0.926, indicating that the gain is not achieved by sacrificing either positive case recall or false positive control. A similar trend is observed on NFHS 5, where the proposed method obtains 0.968±0.003 AUROC and 0.904±0.004 F1 score, exceeding FT Transformer by 1.0 and 1.3 percentage points, respectively, and surpassing XGBoost by 1.5 points in AUROC and 2.0 points in F1 score. These consistent improvements across two independent datasets suggest that the proposed model generalizes better than both tree based and deep tabular baselines.

**Table 4:** Main results on NHANES.

Method	AUROC ↑	F1 score ↑	Sensitivity ↑	Specificity ↑
Logistic Regression (22)	0.903±0.006	0.821±0.009	0.804±0.012	0.872±0.010
Random Forest (23)	0.924±0.005	0.846±0.008	0.833±0.010	0.888±0.009
XGBoost (24)	0.944±0.004	0.872±0.006	0.861±0.008	0.912±0.007
MLP (25)	0.931±0.007	0.854±0.010	0.842±0.011	0.895±0.009
TabNet (26)	0.938±0.006	0.864±0.008	0.851±0.010	0.905±0.008
FT Transformer (27)	0.950±0.004	0.879±0.006	0.867±0.007	0.918±0.006
Proposed Method	0.961±0.003	0.892±0.005	0.883±0.007	0.926±0.005

All results are reported as mean ± standard deviation over five runs.

**Table 5:** Main results on NFHS 5.

Method	AUROC ↑	F1 score ↑	Sensitivity ↑	Specificity ↑
Logistic Regression (22)	0.916±0.005	0.838±0.008	0.821±0.010	0.884±0.009
Random Forest (23)	0.936±0.005	0.862±0.007	0.848±0.009	0.901±0.008
XGBoost (24)	0.953±0.004	0.884±0.006	0.872±0.007	0.920±0.006
MLP (25)	0.941±0.006	0.869±0.008	0.855±0.010	0.907±0.008
TabNet (26)	0.948±0.005	0.878±0.007	0.864±0.008	0.915±0.007
FT Transformer (27)	0.958±0.004	0.891±0.006	0.878±0.007	0.925±0.006
Proposed Method	0.968±0.003	0.904±0.004	0.892±0.006	0.932±0.005

All results are reported as mean ± standard deviation over five runs.

**Table 6:** Efficiency comparison on NHANES and NFHS 5.

Method	Params on NHANES ↓	FLOPs on NHANES ↓	Params on NFHS 5 ↓	FLOPs on NFHS 5 ↓
Logistic Regression (22)	0.00001M	0.00001M	0.00001M	0.00001M
Random Forest (23)	0.028M	0.041M	0.028M	0.041M
XGBoost (24)	0.021M	0.034M	0.021M	0.034M
MLP (25)	0.019M	0.036M	0.019M	0.036M
TabNet (26)	0.041M	0.112M	0.041M	0.112M
FT Transformer (27)	0.087M	0.186M	0.087M	0.186M
Proposed Method	0.061M	0.129M	0.061M	0.129M

The improved results can be attributed to the proposed INMM architecture, which explicitly decomposes the input into dedicated feature extraction branches before feature integration, rather than relying on a single homogeneous encoder. This design enables more structured representation learning even when the final input is compact tabular data. The hierarchical attention mechanism helps the model adaptively reweight informative latent factors, which explains the simultaneous improvement in Sensitivity and Specificity. Compared with FT Transformer, our method is also more efficient, requiring only 0.061M parameters and 0.129M FLOPs vs. 0.087M parameters and 0.186M FLOPs. The proposed method offers a better effectiveness efficiency trade off, making it more suitable for practical child stunting screening scenarios. Taken together, these comparative results provide direct empirical validation of the proposed model. The consistent improvements over both classical machine learning baselines and modern deep tabular baselines on two independent datasets indicate that the proposed INMM architecture is effective for child stunting identification rather than being tailored to a single dataset or comparison setting. Moreover, the repeated run protocol further supports that these gains are stable rather than incidental. Taken together, these results provide empirical support for the effectiveness of the proposed model. The consistent improvements observed across two independent datasets and multiple baseline methods indicate that the performance gains are not specific to a single dataset or configuration.

To further clarify the clinical and deployment relevance of the observed improvement over FT Transformer, an additional discussion was added from the perspective of minimal clinically important difference and computational cost. In child stunting screening, there is no universally accepted minimal clinically important difference for AUROC, because the clinical value of a model depends not only on global discrimination but also on whether additional high risk children can be detected without producing an unacceptable increase in false positives. The approximately 1.0 to 1.1 percentage point AUROC improvement over FT Transformer should not be interpreted as clinically meaningful by AUROC alone. It should be considered together with F1 score, Sensitivity, model size, computational cost, and inference latency. As shown in Table 7, the proposed method improves both discrimination and screening related metrics compared with FT Transformer. On NHANES, Sensitivity increases from 0.867 to 0.883, while Specificity increases from 0.918 to 0.926. On NFHS 5, Sensitivity increases from 0.878 to 0.892, while Specificity increases from 0.925 to 0.932. These results indicate that the gain is not limited to a small AUROC change, but is accompanied by better positive case detection and false positive control, which are more directly relevant to screening practice. From a deployment perspective, the proposed method also uses fewer parameters and FLOPs than FT Transformer, with 0.061M parameters and 0.129M FLOPs compared with 0.087M parameters and 0.186M FLOPs. The measured single sample inference time is also lower than that of FT Transformer on both datasets. The added architectural components of INMM do not introduce a heavier deployment burden than the strongest transformer based baseline. The proposed design provides a more favorable balance between screening performance and computational efficiency. This study does not claim that a 1 percentage point AUROC improvement alone establishes definitive clinical benefit. The results should be interpreted as evidence of improved screening discrimination under an offline supervised evaluation setting. Before real clinical deployment, the model should be externally validated in additional populations, evaluated under site specific operating thresholds, and assessed through prospective screening studies that measure downstream outcomes such as missed stunting cases, unnecessary referrals, workload, and cost. Under the current experimental setting, the additional complexity of the proposed method is justified because it improves clinically relevant classification metrics while requiring fewer parameters, fewer FLOPs, and lower inference latency than FT Transformer.

**Table 7:** Clinical relevance and deployment efficiency comparison between FT Transformer and the proposed method.

Method	Dataset	AUROC ↑	F1 score ↑	Sensitivity ↑	Specificity ↑	Params ↓	FLOPs ↓	Inference time ↓
FT Transformer	NHANES	0.950±0.004	0.879±0.006	0.867±0.007	0.918±0.006	0.087M	0.186M	0.082 ms
Proposed Method	NHANES	0.961±0.003	0.892±0.005	0.883±0.007	0.926±0.005	0.061M	0.129M	0.061 ms
FT Transformer	NFHS 5	0.958±0.004	0.891±0.006	0.878±0.007	0.925±0.006	0.087M	0.186M	0.083 ms
Proposed Method	NFHS 5	0.968±0.003	0.904±0.004	0.892±0.006	0.932±0.005	0.061M	0.129M	0.062 ms

Inference time denotes the average single sample forward inference latency measured on the same hardware environment.

To further address the relevance of the experimental datasets and verify the generalizability of the proposed method on additional child health survey data, supplementary comparative experiments were conducted on DHS Program and UNICEF MICS. These two datasets are closely aligned with the objective of child stunting identification because they provide child level anthropometric variables that can be used to generate WHO based stunting labels. The same preprocessing pipeline, input variables, stratified data split, training strategy, validation based model selection, and five run statistical protocol were used for all methods. The results are summarized in Table 8. The supplementary results show that the proposed method maintains consistent advantages on both additional datasets. On DHS Program, the proposed method achieves an AUROC of 0.966±0.003 and an F1 score of 0.901±0.004, outperforming FT Transformer by 1.1 percentage points in AUROC and 1.4 percentage points in F1 score. On UNICEF MICS, the proposed method obtains an AUROC of 0.967±0.003 and an F1 score of 0.903±0.004, exceeding FT Transformer by 1.0 percentage points and 1.3 percentage points, respectively. The proposed method also improves Sensitivity and Specificity on both datasets, indicating that the performance gain is not achieved by sacrificing either positive case detection or false positive control. These supplementary experiments provide additional evidence that the proposed INMM is effective on more relevant child anthropometric survey data and that the observed improvements are consistent with the objective and scope of child stunting identification.

**Table 8:** Supplementary comparative results on DHS Program and UNICEF MICS.

Dataset	Method	AUROC ↑	F1 score ↑	Sensitivity ↑	Specificity ↑
DHS Program	Logistic Regression	0.909±0.005	0.831±0.008	0.815±0.010	0.879±0.009
DHS Program	Random Forest	0.931±0.005	0.857±0.007	0.843±0.009	0.899±0.008
DHS Program	XGBoost	0.949±0.004	0.879±0.006	0.867±0.007	0.917±0.006
DHS Program	MLP	0.938±0.006	0.864±0.008	0.850±0.009	0.904±0.008
DHS Program	TabNet	0.945±0.005	0.874±0.007	0.861±0.008	0.912±0.007
DHS Program	FT Transformer	0.955±0.004	0.887±0.006	0.874±0.007	0.923±0.006
DHS Program	Proposed Method	0.966±0.003	0.901±0.004	0.890±0.006	0.931±0.005
UNICEF MICS	Logistic Regression	0.912±0.005	0.834±0.008	0.818±0.010	0.882±0.009
UNICEF MICS	Random Forest	0.934±0.005	0.860±0.007	0.846±0.009	0.902±0.008
UNICEF MICS	XGBoost	0.951±0.004	0.881±0.006	0.869±0.007	0.919±0.006
UNICEF MICS	MLP	0.940±0.006	0.867±0.008	0.853±0.009	0.906±0.008
UNICEF MICS	TabNet	0.947±0.005	0.877±0.007	0.864±0.008	0.914±0.007
UNICEF MICS	FT Transformer	0.957±0.004	0.890±0.006	0.877±0.007	0.924±0.006
UNICEF MICS	Proposed Method	0.967±0.003	0.903±0.004	0.891±0.006	0.932±0.005

All results are reported as mean ± standard deviation over five runs.

#### 4.5.2 Ablation study

We perform an ablation study to examine which components of the proposed method are responsible for the final performance gains on NHANES and NFHS 5. The design follows the method summary and focuses on two complementary aspects. We conduct a main module level ablation by progressively removing or simplifying key components derived from the proposed pipeline, including feature extraction, feature fusion, hierarchical attention, and the nonlinear prediction head. This setting is necessary because the full model is built as a sequence of explicit stages, and the contribution of each stage should be verified under the same supervised binary classification objective, preprocessing protocol, data split, and statistical setting. We perform a compact sensitivity and robustness study with three sensitivity factors and one robustness factor that are most consistent with the task and data type. The sensitivity factors are hidden dimension, dropout rate, and weight decay, because they directly affect representation capacity, regularization strength, and optimization stability in tabular binary classification. The robustness factor is missing feature perturbation at test time, because the task relies on a small set of clinical tabular variables and real world screening data are vulnerable to incomplete measurements. All ablation results are reported with the same evaluation metrics, repeated run protocol, and significance aware analysis as the main comparison experiments, ensuring fair and reproducible interpretation.

The ablation results in Table 9 confirm that each component of the proposed method contributes non redundantly to the final gains on both datasets. On NHANES, the full model achieves an AUROC of 0.961±0.003 and an F1 score of 0.892±0.005, compared with 0.929±0.007 and 0.851±0.010 for the linear predictor only variant, yielding absolute improvements of 3.2 and 4.1 percentage points, respectively. Introducing the proposed feature extraction module already raises performance to 0.944 AUROC and 0.871 F1 score, suggesting that explicit branch wise encoding is effective even for compact child level tabular inputs. Adding feature fusion further improves NHANES performance to 0.952 AUROC and 0.882 F1 score, which indicates that the fused representation learned from the structured branches captures complementary information better than isolated latent features. When hierarchical attention is included but the nonlinear classifier is replaced by a linear head, performance increases to 0.956 AUROC and 0.887 F1 score; however, it still remains below the full model, showing that attention alone is insufficient without the proposed nonlinear prediction head. To further examine whether body mass index provides useful information beyond its source measurements, an additional feature level ablation was conducted by removing BMI from the input variables while keeping the full model architecture and all training settings unchanged. In this setting, each child sample was represented only by age, sex, height, and weight. As shown in Table 9, removing BMI decreases the NHANES AUROC from 0.961 to 0.954 and the F1 score from 0.892 to 0.883. On NFHS 5, AUROC decreases from 0.968 to 0.962, and F1 score decreases from 0.904 to 0.895. Although BMI is mathematically derived from height and weight, it provides a compact and standardized summary of relative body mass with respect to height. The consistent performance decrease after removing BMI indicates that BMI is not merely a redundant duplicate in the proposed framework, but contributes complementary derived anthropometric information for child stunting identification. A similar pattern appears on NFHS 5, where the full model reaches 0.968±0.003 AUROC and 0.904±0.004 F1 score, improving over the linear predictor only setting by 2.8 and 3.8 percentage points, respectively. Notably, removing hierarchical attention causes both Sensitivity and Specificity to decline, for example from 0.883/0.926 to 0.870/0.920 on NHANES, which suggests that the proposed attention mechanism improves the balance between positive case detection and false positive suppression by adaptively emphasizing informative latent factors. The results support that the superiority of the proposed method comes from the joint effect of the proposed feature extraction architecture, fusion strategy, hierarchical attention mechanism, nonlinear prediction head, and the inclusion of BMI as a derived anthropometric feature rather than from any single module alone.

**Table 9:** Main module and feature ablation results on NHANES and NFHS 5.

Variant	NHANES				NFHS 5			
	AUROC ↑	F1 score ↑	Sensitivity ↑	Specificity ↑	AUROC ↑	F1 score ↑	Sensitivity ↑	Specificity ↑
Linear predictor only	0.929±0.007	0.851±0.010	0.838±0.012	0.894±0.010	0.940±0.006	0.866±0.009	0.852±0.011	0.906±0.009
Feature extraction + classifier	0.944±0.005	0.871±0.007	0.858±0.009	0.911±0.008	0.954±0.005	0.885±0.007	0.871±0.008	0.919±0.007
Feature extraction + fusion + classifier	0.952±0.004	0.882±0.006	0.870±0.008	0.920±0.006	0.961±0.004	0.894±0.006	0.881±0.007	0.927±0.006
Feature extraction + fusion + attention + linear head	0.956±0.004	0.887±0.006	0.878±0.007	0.921±0.006	0.965±0.003	0.899±0.005	0.888±0.006	0.929±0.005
Full model without BMI	0.954±0.004	0.883±0.006	0.872±0.008	0.921±0.006	0.962±0.004	0.895±0.005	0.884±0.007	0.927±0.006
Full model	0.961±0.003	0.892±0.005	0.883±0.007	0.926±0.005	0.968±0.003	0.904±0.004	0.892±0.006	0.932±0.005

All results are reported as mean ± standard deviation over five runs.

Table 10 further shows that the proposed model is stable under moderate hyperparameter variation and remains reasonably robust under incomplete input conditions. Using a hidden dimension of 64 gives the best results on both NHANES and NFHS 5, while reducing it to 32 leads to only a limited drop from 0.961 to 0.954 in AUROC on NHANES and from 0.968 to 0.963 on NFHS 5, indicating that the model does not rely on excessive representation width. Increasing the hidden dimension to 128 also does not provide further benefit, which suggests that the proposed architecture already captures sufficient task relevant information with a moderate latent size. A similar trend is observed for regularization settings. Changing dropout from 0.5 to 0.3 decreases NHANES AUROC by only 0.3 percentage points and NFHS 5 AUROC by 0.2 percentage points, while modifying weight decay from $1\times 10^{-4}$ to $1\times 10^{-5}$ results in similarly small variations. These results indicate that the proposed INMM based design is not overly sensitive to minor optimization changes, which is desirable for reproducible deployment. For the missing feature perturbation setting, the perturbation was applied only to the held out test set, while the training and validation data remained unchanged. For each test sample, one feature was uniformly selected at random from the five input variables, namely age, sex, height, weight, and BMI, and the selected feature value was replaced by zero after z score normalization. Since the continuous variables were standardized using statistics from the training split, replacing a continuous feature with zero corresponds to imputing the training set mean in the normalized feature space. For the numerically encoded sex variable, zero replacement was used as the same controlled masking operation to simulate an unavailable structured input field. The model was not retrained under this setting, and the same trained model was directly evaluated on the perturbed test samples. This protocol was repeated under the same five random seeds as the main experiments, and the reported results represent the mean and standard deviation across the five runs. Under this missing feature perturbation protocol, performance degrades gracefully rather than collapsing, with NHANES AUROC dropping from 0.961 to 0.946 and NFHS 5 AUROC dropping from 0.968 to 0.955. The corresponding F1 score remains at 0.874 and 0.887, respectively, showing that the proposed method preserves useful screening capability even when one randomly selected demographic or anthropometric variable is unavailable at test time. This robustness can be attributed to the proposed fusion and attention mechanisms, which reduce over reliance on any single input variable by redistributing importance across latent representations. The ablation, sensitivity, and robustness analyses further validate the proposed model from three complementary perspectives: component effectiveness, training stability, and tolerance to incomplete input conditions. These results strengthen the conclusion that the reported performance gains are attributable to the proposed architecture itself rather than to accidental configuration choices. These analyses also provide insights into the model behavior under different perturbations and can be interpreted as an empirical error analysis of the proposed method.

**Table 10:** Sensitivity and robustness analysis on NHANES and NFHS 5.

Setting	NHANES				NFHS 5			
	AUROC ↑	F1 score ↑	Sensitivity ↑	Specificity ↑	AUROC ↑	F1-score ↑	Sensitivity ↑	Specificity ↑
Hidden dimension = 32	0.954±0.005	0.884±0.007	0.872±0.009	0.921±0.007	0.963±0.004	0.896±0.006	0.883±0.008	0.928±0.006
Hidden dimension = 64	0.961±0.003	0.892±0.005	0.883±0.007	0.926±0.005	0.968±0.003	0.904±0.004	0.892±0.006	0.932±0.005
Hidden dimension = 128	0.959±0.004	0.890±0.006	0.880±0.008	0.924±0.006	0.967±0.003	0.902±0.005	0.890±0.007	0.931±0.005
Dropout = 0.3	0.958±0.004	0.888±0.006	0.879±0.008	0.923±0.006	0.966±0.004	0.901±0.005	0.889±0.007	0.930±0.005
Dropout = 0.5	0.961±0.003	0.892±0.005	0.883±0.007	0.926±0.005	0.968±0.003	0.904±0.004	0.892±0.006	0.932±0.005
Weight decay = $1\times 10^{-5}$	0.959±0.004	0.889±0.006	0.880±0.008	0.924±0.006	0.967±0.004	0.902±0.005	0.890±0.007	0.931±0.005
Weight decay = $1\times 10^{-4}$	0.961±0.003	0.892±0.005	0.883±0.007	0.926±0.005	0.968±0.003	0.904±0.004	0.892±0.006	0.932±0.005
Missing feature perturbation	0.946±0.005	0.874±0.007	0.861±0.009	0.913±0.007	0.955±0.004	0.887±0.006	0.874±0.008	0.920±0.006

All results are reported as mean ± standard deviation over five runs.

### 4.6 Supplementary evaluation of ANIS

To further evaluate the practical utility of the Adaptive Nutritional Intervention Strategy, an additional screening oriented experiment was conducted on both NHANES and NFHS 5. In this experiment, ANIS operates as a post prediction risk prioritization module based on the latent nutritional representations and stunting probabilities generated by INMM. After INMM predicts the stunting probability for each child, ANIS computes an additional priority score by combining the predicted probability with the latent risk representation learned from the structured demographic and anthropometric features. Children are then ranked according to the resulting priority scores. The purpose of this experiment is to examine whether the proposed strategy can improve the identification priority of high risk children under limited screening capacity. For the ith child, the priority score is defined as (Equation 20):

$$S_{i}=\alpha p_{i}+(1-\alpha )r_{i},$$ (20)

where $p_{i}$ denotes the stunting probability predicted by INMM, $r_{i}$ denotes the normalized latent risk score derived from the aggregated feature representation, and α is a weighting coefficient selected on the validation set. Different from the binary classification metrics used in the main experiments, this evaluation focuses on ranking based screening performance. Three metrics are adopted, including Precision at top K, Recall at top K, and normalized discounted cumulative gain. Precision at top K evaluates the proportion of truly stunted children among the highest priority samples. Recall at top K measures the proportion of all stunted children captured within the highest ranked group. Normalized discounted cumulative gain evaluates whether high risk children are ranked near the top of the screening list. Table 11 presents the screening priority results on NHANES and NFHS 5. Compared with directly ranking children using only the prediction probability generated by INMM, the proposed ANIS strategy achieves consistent improvement across all ranking based metrics. On NHANES, Recall at top K increases from 0.846 to 0.872, while normalized discounted cumulative gain improves from 0.891 to 0.913. Similar improvements are observed on NFHS 5, where Recall at top K increases from 0.861 to 0.887 and normalized discounted cumulative gain increases from 0.904 to 0.921. These results indicate that the latent nutritional representation learned by INMM provides additional complementary information for risk prioritization beyond the final classification probability alone. The experimental results suggest that ANIS can improve high risk child prioritization under resource constrained screening settings. This capability is particularly important for practical child health screening scenarios in which early attention should be allocated preferentially to children with elevated stunting risk. The observed improvements further demonstrate that the proposed framework can support not only binary stunting identification but also screening oriented risk ordering and prioritization.

**Table 11:** Supplementary evaluation of ANIS for screening oriented risk prioritization.

Method	NHANES			NFHS 5		
	Precision@K ↑	Recall@K ↑	NDCG ↑	Precision@K ↑	Recall@K ↑	NDCG ↑
INMM probability ranking	0.831±0.008	0.846±0.007	0.891±0.006	0.845±0.007	0.861±0.006	0.904±0.005
INMM + ANIS	0.856±0.006	0.872±0.006	0.913±0.005	0.869±0.006	0.887±0.005	0.921±0.004

All results are reported as mean ± standard deviation over five runs.

The bold values indicates the optimal values.

## 5 Conclusions and future work

In this study, we proposed a deep learning based framework for monitoring children’s nutritional status for child stunting identification. Different from a broad structured health assessment setting, the final framework was formulated as a supervised binary classification task based on routinely available structured child level variables, including age, sex, height, weight, and body mass index. Under this task definition, we developed the Intelligent Nutritional Monitoring Model (INMM), which combines branch wise feature extraction, feature fusion, hierarchical attention, and a nonlinear prediction head to learn discriminative latent representations from tabular demographic and anthropometric data. Extensive experiments on two public health survey datasets, NHANES and NFHS 5, demonstrated that the proposed method achieved the strongest overall classification performance among all compared baselines. In particular, the model consistently outperformed traditional machine learning methods, mainstream deep learning models, lightweight tabular architectures, and transformer based tabular baselines in terms of AUROC, F1 score, Sensitivity, and Specificity, while maintaining a favorable effectiveness efficiency trade off. Additional ablation and sensitivity analyses further confirmed that the proposed feature extraction, fusion, attention, and prediction design all contributed non redundantly to the final performance and that the framework remained stable under moderate hyperparameter variation and incomplete feature perturbation.

Despite these promising results, this study still has several limitations. The current framework relies on only a small set of structured demographic and anthropometric variables. Although such inputs are practical and widely available, they may not fully capture the broader clinical, behavioral, and socioeconomic factors associated with child stunting. The experiments were conducted on two public survey datasets under an offline supervised evaluation setting. The generalizability of the proposed framework to other populations, healthcare systems, and real world screening environments still requires further external validation. Another important limitation is that the Sensitivity of the proposed method, although higher than that of the compared baselines, remains modest for a screening oriented child stunting identification task. The proposed method achieves Sensitivity values of 0.883 on NHANES and 0.892 on NFHS 5, which means that approximately 11.7% and 10.8% of stunted children may still be missed under the operating threshold used in the experiments. This false negative rate is clinically important, because missed stunting cases may delay further growth assessment, nutritional counseling, and public health follow up. The proposed framework should not be interpreted as a standalone diagnostic tool, but rather as a data driven decision support method for preliminary screening and risk prioritization. In practical deployment, the decision threshold should be selected according to the screening objective and local resource constraints rather than fixed only by the default classification rule. When the priority is to reduce missed stunting cases, the probability threshold can be lowered on a validation set to increase Sensitivity, although this may reduce Specificity and increase the number of children referred for further assessment. When follow up resources are limited, a higher threshold may be used to reduce false positives at the cost of missing more true stunting cases. A clinically appropriate threshold should therefore be determined through validation based threshold calibration, receiver operating characteristic analysis, precision recall analysis, and consultation with public health practitioners. Third, the present study focuses on stunting identification rather than direct intervention optimization or longitudinal outcome forecasting, so its utility should be interpreted primarily as a data driven screening support tool. Future work will extend the framework in several directions. One important direction is to incorporate richer child health information, such as dietary, household, maternal, and longitudinal growth variables, to improve predictive completeness while preserving practical usability. Another direction is to evaluate the framework on more diverse external cohorts and prospective screening scenarios to assess robustness and deployment potential. Future work should also report model performance under multiple operating points, especially high Sensitivity settings suitable for child growth screening, and prospectively evaluate threshold adjustment strategies in real screening workflows. The adaptive decision support strategy can be further refined and validated as a practical extension for risk prioritization and follow up screening, so that the overall framework can better support scalable child health monitoring in real world settings.

## Data Availability

The original contributions presented in the study are included in the article/Supplementary Material, further inquiries can be directed to the corresponding author/s.
